# Serotonin Antagonism Improves Platelet Inhibition in Clopidogrel Low-Responders after Coronary Stent Placement: An *In Vitro* Pilot Study

**DOI:** 10.1371/journal.pone.0032656

**Published:** 2012-02-27

**Authors:** Daniel Duerschmied, Ingo Ahrens, Maximilian Mauler, Christoph Brandt, Stefanie Weidner, Christoph Bode, Martin Moser

**Affiliations:** Department of Cardiology and Angiology, University Medical Center Freiburg, Freiburg, Germany; Heart Center Munich, Germany

## Abstract

Increased residual platelet reactivity remains a burden for coronary artery disease (CAD) patients who received a coronary stent and do not respond sufficiently to treatment with acetylsalicylic acid and clopidogrel. We hypothesized that serotonin antagonism reduces high on-treatment platelet reactivity. Whole blood impedance aggregometry was performed with arachidonic acid (AA, 0.5 mM) and adenosine diphosphate (ADP, 6.5 µM) in addition to different concentrations of serotonin (1–100 µM) in whole blood from 42 CAD patients after coronary stent placement and 10 healthy subjects. Serotonin increased aggregation dose-dependently in CAD patients who responded to clopidogrel treatment: After activation with ADP, aggregation increased from 33.7±1.3% to 40.9±2.0% in the presence of 50 µM serotonin (p<0.05) and to 48.2±2.0% with 100 µM serotonin (p<0.001). The platelet serotonin receptor antagonist ketanserin decreased ADP-induced aggregation significantly in clopidogrel low-responders (from 59.9±3.1% to 37.4±3.5, p<0.01), but not in clopidogrel responders. These results were confirmed with light transmission aggregometry in platelet-rich plasma in a subset of patients. Serotonin hence increased residual platelet reactivity in patients who respond to clopidogrel after coronary stent placement. In clopidogrel low-responders, serotonin receptor antagonism improved platelet inhibition, almost reaching responder levels. This may justify further investigation of triple antiplatelet therapy with anti-serotonergic agents.

## Introduction

In myocardial infarction - the critical event in coronary artery disease (CAD) - plaque rupture initiates platelet activation resulting in atherothrombotic coronary artery occlusion [1]. Percutaneous coronary intervention with stent placement is the standard of care treatment for patients with critical CAD [2], [3]. Implantation of a coronary stent further stimulates the adhesion and activation of platelets, which makes a highly efficient inhibition of platelet activation mandatory until endothelialization of the stent is complete [4]. Current guidelines recommend dual antiplatelet therapy for these patients, consisting of the lifelong administration of acetylsalicylic acid (ASA, aspirin - cyclooxygenase inhibition) in combination with an adenosine diphosphate (ADP) P2Y_12_ receptor antagonist for the first 12 months [2], [3]. In acute coronary syndrome the newer P2Y_12_ receptor antagonists ticagrelor and prasugrel are preferred, but clopidogrel remains the standard substance when ticagrelor or prasugrel are contraindicated or unavailable and in patients undergoing elective stenting.

An important clinical problem is hypo-responsiveness to clopidogrel [5], [6]. Clopidogrel is a prodrug and must be metabolized in intestines and liver to produce an active metabolite that binds the P2Y_12_ receptor [7]. In March 2010, the Food and Drug Administration added a boxed warning to the label of clopidogrel to highlight its reduced effectiveness in poor metabolizers. Approximately 30% of patients are considered poor responders as evaluated by ADP-induced platelet aggregation [8], [9], [10]. Apart from absorption and receptor reactivity, genetic and drug-induced variations in cytochrome P450 activity are responsible for the inter-individual variability in clopidogrel responsiveness [11]. High on-treatment platelet reactivity to ADP is associated with adverse clinical event occurrence, but the wide variety of definitions of residual platelet reactivity has only recently been addressed by a consensus statement [12]. To date, it is not clear which measure of platelet reactivity and which cut-off points should be used.

Light transmission aggregometry (LTA) is largely considered as the gold standard to evaluate platelet function with respect to pharmacological platelet inhibition, but is poorly standardized [12], [13]. For LTA, platelet-rich plasma (PRP) is prepared by centrifugation and stimulated by adding a platelet agonist. The increase of transmitted light is proportional to the formation of aggregates under stirring conditions. The level of platelet aggregation in LTA predicted the early outcome after elective coronary stent placement in 802 patients treated with clopidogrel [14]. Whole blood impedance aggregometry, like e.g. with the point-of-care assay Multiplate® measures the increase in impedance due to attachment of platelets to electrodes [15]. A study with 416 patients undergoing coronary stent placement suggested that multiple electrode aggregometry (MEA) with Multiplate is equivalent to other assays in identifying patients at risk for stent thrombosis [16].

Serotonin is a weak platelet activator promoting hemostasis by enhancing platelet alpha granule release and by enhancing plasmatic coagulation [17], [18], [19], [20]. Together with ADP, serotonin is stored in platelet dense granules (at millimolar concentrations) and released upon platelet activation, locally reaching micromolar levels at sites of coronary atherothrombosis [21], [22], [23]. Released serotonin is also a potent vasoconstrictor of coronary arteries with damaged endothelium [24]. Serotonin antagonism as well as platelet serotonin depletion by treatment with selective serotonin reuptake inhibitors have both been associated with a reduction in arterial thrombogenicity [17], [25], [26]. Therefore, serotonin antagonism in addition to established therapies might be a promising approach to improve the treatment of CAD patients. This concept was proposed as triple antiplatelet therapy, but to our knowledge has not yet been evaluated systematically. In consequence, we hypothesized that serotonin antagonism may reduce high on-treatment platelet reactivity in ASA- and clopidogrel-treated patients. We used MEA in whole blood from CAD patients who had received a coronary stent within the past 1–3 days to test this hypothesis and confirmed the results with LTA. We found first that addition of exogenous serotonin increased platelet aggregation in blood from patients who responded well to ASA and clopidogrel treatment. Secondly, *in vitro* serotonin receptor antagonism with ketanserin reduced high on-treatment platelet reactivity in clopidogrel low-responders.

## Materials and Methods

### Patient enrollment and ethics statement

This pilot study examined the *ex vivo* effects of serotonin and a serotonin receptor antagonist on platelets from patients who were treated with ASA and clopidogrel after coronary stent placement. The protocol complied with the Declaration of Helsinki and was approved by the Ethics Committee of the University of Freiburg, Germany. The study was planned prospectively. Participants were considered eligible for enrollment if they were >18 years of age. CAD patients gave verbal informed consent for additional ex vivo measurements with routinely acquired platelet aggregometry blood samples (which was documented in a log sheet) and written consent was acquired retrospectively to comply with ethical guidelines. Healthy control subjects and CAD patients donating an additional blood sample for LTA gave written informed consent before sample acquisition. CAD patients receiving ASA and clopidogrel were recruited consecutively from the local Department of Cardiology from November 4^th^, 2009 through December 2^nd^, 2010 and CAD patients receiving ASA only were recruited from November 20^th^, 2011 through December 29^th^, 2011. Depression and current medication with serotonin reuptake inhibitors were exclusion criteria. Thrombocytopenia ≤100,000/mm^3^ was also an exclusion criterion. CAD patients had received a loading dose of 600 mg clopidogrel and at least 300 mg ASA prior to coronary angiography according to standard operating procedure at the local institution. Patients then received 75 mg clopidogrel and 100 mg ASA once daily and compliance was documented. A blood sample was taken on day 1–3 after stent placement, while the patients were still hospitalized. Acute coronary syndrome patients who had received treatment with a glycoprotein IIb/IIIa inhibitor were excluded. CAD patients treated with ASA only had not undergone coronary angiography and were not exposed to clopidogrel prior to blood sampling. Control subjects were healthy and without current medication. Control subjects were not included if they had taken ASA or any other non-steroidal anti-inflammatory drug within the prior 7 days.

A power calculation for an alpha or 0.05 yielded a necessary sample size of at least 10 per group to detect a difference in mean aggregation of 9.5% with an assumed standard deviation of 7% with a power of 80% (G*Power 3.0.10, Heinrich Heine University, Duesseldorf, Germany). Between 15 and 40% of patients were considered poor responders to clopidogrel treatment in several studies applying similar criteria as in our study (see below) [8], [9], [10]. We therefore envisioned including 45 CAD patients in order to have a group of approximately 10 clopidogrel low-responders. Two CAD patients were excluded from the study because the platelet aggregation response to TRAP was below the pre-defined threshold (see below) and 1 CAD patient withdrew informed consent.

### Materials

Serotonin hydrochloride, ketanserin, sarpogrelate hydrochloride, and Prostaglandin (Pg) E_1_ were obtained from Sigma-Aldrich (Munich, Germany). Thrombin receptor activating peptide-6 (TRAP), arachidonic acid (AA), and ADP were obtained from Verum Diagnostica (Munich, Germany). Blood collection tubes were purchased from Sarstedt (Nuernbrecht, Germany).

### Sample acquisition

A 5.2 ml blood sample was taken from all study participants for multiple electrode aggregometry (MEA) and anticoagulated with 0.045 mg/ml hirudin. For LTA, 30 ml of blood were taken from all control subjects and a third of the enrolled CAD patients (randomly chosen) and anticoagulated with 0.011 mol/l trisodium citrate. MEA and LTA were performed within 2 h after sample acquisition.

### Whole blood impedance aggregometry

Hirudinized blood was diluted with an equal volume of normal saline and kept under stirring conditions at 37°C in a Multiplate analyzer (Verum Diagnostica, Munich, Germany). Samples were then incubated with the indicated concentrations of serotonin, ketanserin, or vehicle (0.001% ascorbic acid and 1∶1,000 DMSO) for 1 min. Finally, the platelet agonist was added at standardized final concentrations: 32 µM TRAP, 0.5 mM AA, 6.5 µM ADP, or vehicle. MEA curves from two electrode pairs were recorded for each sample and the area under the curves (AUC) was averaged. Blood samples showing a response to TRAP of ≤450 AU/min did not meet the criteria for a sufficient positive platelet aggregation control and were excluded from further analysis (4.4% of the examined samples; reasons may have been delayed sample analysis or unreported use of other antiplatelet agents). To ensure optimal comparability of the effects of serotonergic stimulation, the aggregation responses were normalized to the response to TRAP in all samples from each study participant (denoted as % of TRAP).

### Definition of low-responder status

Definition of clopidogrel low-responder status remains a matter of debate and several groups have suggested different approaches, resulting in a consensus statement that provided definitions for different platelet function tests in 2010 [12], [27]. In accordance with this statement we defined clopidogrel low-responsiveness as an area under the MEA curve of ≥468 AU/min after ADP stimulation [12], [27]. This corresponded to ≥48% of maximal stimulation by thrombin receptor activation in all low-responders (while all responders were <48%). To ensure comparability, low-responsiveness in LTA was therefore also defined as maximal aggregation ≥48% of maximal response to thrombin receptor activation.

### Light transmission aggregometry

LTA was chosen as a gold standard for comparison with MEA findings using the same platelet agonist concentrations as described above. One third of the recruited CAD patients treated with ASA and clopidogrel (n = 15) and all CAD patients treated with ASA only and control subjects were asked to donate an additional 30 ml of blood, which was refused by 2 CAD patients. Platelet-rich plasma (PRP) was obtained from citrated blood by centrifugation at 100 g for 10 min and adjusted to a platelet count of 2.5×10^8^/ml with platelet-poor plasma (obtained by centrifugation at 1,000 g for 5 min). PRP was re-calcified with 1 mM CaCl_2_ and incubated with serotonin, ketanserin, or vehicle under stirring conditions for 1 min at 37°C. Platelet agonists were added as described above in MEA and LTA traces were recorded for 15 min. Maximal aggregation was defined as % light transmission compared to platelet-poor plasma.

### Measurement of serotonin release

After performing MEA, the supernatant (hirudinized whole blood devoid of platelet aggregates in case of platelet activation) was aspirated from the Multiplate cuvettes in a subset of 13 CAD patients. To avoid additional platelet activation by the following centrifugation steps, blood was incubated with 5 mM EDTA and 4 µg/ml PgE_1_ for 5 min. Cell-free plasma was prepared by centrifugation at 1,000 g for 5 min followed by 16,000 g for 5 min and stored at −80°C. Serotonin concentration was measured with a high-sensitivity ELISA (Labor Diagnostika Nord, Hamburg, Germany).

### Statistical Analysis

Results are reported as mean and standard error of the mean. Contingency tables for clinical characteristics were analyzed with Fisher's exact test. One-way ANOVA followed by Bonferroni's multiple comparison test or unpaired t-test were used to compare quantification variables between three or two groups, respectively. All statistical tests were two-sided and a *P* value of <0.05 was interpreted to denote statistical significance. The statistical analysis was performed with GraphPad Prism 4.0 (GraphPad Software Inc., La Jolla, CA, USA).

## Results

### Study subjects

The 42 included CAD patients who were treated with ASA and clopidogrel showed a typical risk profile and 57% presented with acute coronary syndrome. Two thirds of these patients received at least one drug-eluting stent. There were no significant differences in the frequency of smoking, hypertension, dyslipidemia, Diabetes mellitus and ACS between clopidogrel responders and low-responders (Table 1). The group of CAD patients who were treated with ASA only was well matched with the group of CAD patients treated with ASA and clopidogrel. Control subjects were younger and healthy.

**Table 1:** Baseline clinical characteristics of CAD patients treated with ASA only and CAD patients treated with ASA and clopidogrel.

Variable/parameter	ASA only(n = 9)	Clopidogrel low-responders(n = 12)	Clopidogrel responders(n = 30)	*P* (ASA only vs. ASA+clopidogrel)	*P* (clopidogrel low-responders vs. responders)
Age (years) Mean ± SD	73±9	73±14	67±12	0.274	0.144
Male gender	7 (78)	9 (75)	18 (60)	0.699	0.485
Smokers	4 (44)	3 (25)	15 (50)	1.0	0.180
Hypertension	5 (56)	7 (58)	23 (77)	0.436	0.274
Dyslipidemia	3 (33)	6 (50)	16 (53)	0.465	1.0
Diabetes mellitus	3 (33)	1 (8)	4 (13)	0.137	1.0
Acute coronary syndrome	0	6 (50)	18 (60)		0.732
Drug-eluting stent	0	9 (75)	20 (67)		0.722
Bare-metal stent	0	4 (33)	10 (33)		1.0

Healthy control subjects (n = 10) were 34±4 years old, 5 were male, 1 smoked and none suffered from the mentioned diseases. *P* values for the comparison of CAD patients with ASA only vs. all CAD patients with ASA+clopidogrel and for the comparison of clopidogrel low-responders vs. responders. Number of patients (%), unless otherwise indicated.

### Platelet aggregation traces

**Figure 1:**
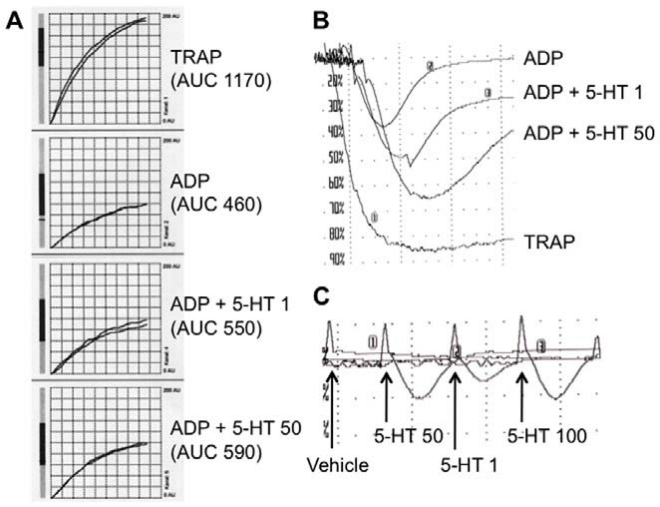
Representative aggregation traces. (A) Exemplary multiple electrode aggregomety traces in whole blood from a clopidogrel responder after adding the depicted agonists (AUC denotes area under the curve). (B) Light transmission aggregometry (LTA) with platelet-rich plasma and the depicted agonists. (C) LTA with vehicle or serotonin only. The following agonist concentrations were used: 32 µM TRAP, 6.5 µM ADP, 1, 50, or 100 µM serotonin.

Incubation with 30 µM TRAP (used as the reference positive control in this study) resulted in an increase in impedance in MEA (reflected by the AUC, Fig. 1A) and induced visible platelet aggregate formation under stirring conditions in LTA (increasing light transmission, Fig. 1B). The increase in light transmission after treatment with 6.5 µM ADP was reversible, especially in clopidogrel-treated patients, as has consistently been reported before [28], [29]. Serotonin at different concentrations alone induced only small increases in light transmission that were also quickly reversible (Fig. 1C), but enhanced the response to ADP in both, MEA and LTA.

### Serotonin increases residual platelet reactivity

**Figure 2:**
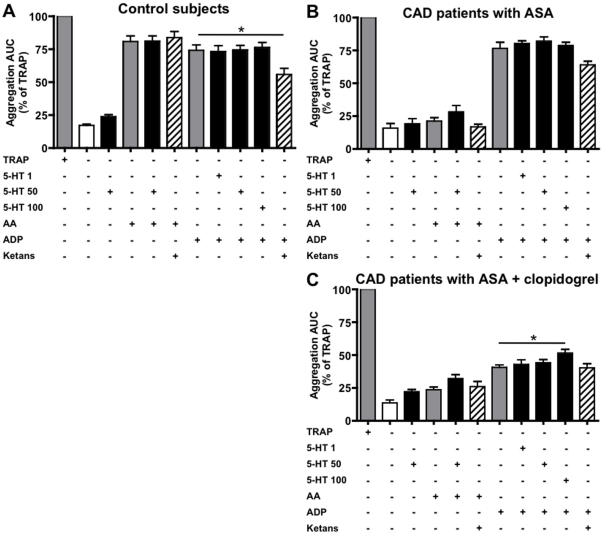
Serotonin increases platelet reactivity in whole blood impedance aggregometry. Whole blood from healthy control subjects (A, n = 10), coronary artery disease (CAD) patients treated with ASA alone (B, n = 9), and the total study cohort of CAD patients treated with ASA and clopidogrel (C, n = 42) after incubation with the depicted substances. The following agonist/antagonist concentrations were used: 32 µM TRAP, 0.5 mM AA, 50 µM serotonin, 10 µM ketanserin. * p<0.05 in ANOVA followed by Bonferroni's multiple comparison test.

To test whether exogenous serotonin influenced residual platelet reactivity in patients treated with ASA and clopidogrel, we measured the AUC in MEA after stimulation with the depicted agonists in all study subjects. In healthy control subjects, incubation with vehicle, serotonin (50 µM), AA (0.5 mM), and ADP (6.5 µM) induced 17.1±1.0%, 23.8±1.4%, 80.8±4.2%, and 74.1±4.2% of maximal (i.e. TRAP-induced) aggregation, respectively (Figure 2A). Serotonin did not further enhance AA- or ADP-induced aggregation and ketanserin did not inhibit AA-induced aggregation. On the contrary, ketanserin significantly decreased ADP-induced aggregation by 24.8% in this group. Similar results were seen in CAD patients who were treated with ASA only (Fig. 2B), except for the well-inhibited response to AA. Ketanserin decreased ADP-induced aggregation by 16.4% (not significant).

In the entire cohort of ASA- and clopidogrel-treated CAD patients, vehicle-, serotonin-, AA-, and ADP-induced aggregation was 13.6±2.2%, 22.2±1.6%, 23.5±2.1%, and 40.6±1.9%, respectively (Figure 2C). Addition of 50 µM serotonin resulted in a non-significant increase in AA-induced aggregation to 32.2±2.9%. ADP-induced aggregation increased dose-dependently after addition of serotonin (up to 51.6±2.7% with 100 µM serotonin, p<0.05), while ketanserin did not induce significant changes. Adding ketanserin to serotonin-treated samples abolished the enhancement of ADP-induced aggregation by serotonin entirely (not shown). These findings indicate that serotonin enhances residual platelet reactivity in ASA- and clopidogrel-treated patients and that serotonin antagonism decreases ADP-induced aggregation in control subjects.

### Serotonin increases ADP-induced platelet aggregation in clopidogrel responders

**Figure 3:**
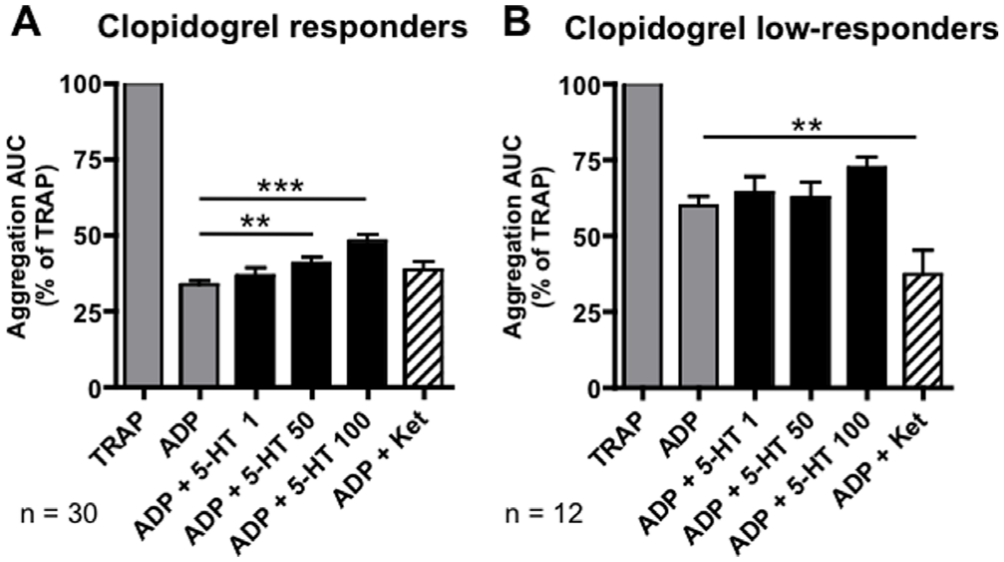
Serotonin enhances ADP-induced aggregation in clopidogrel responders and ketanserin reverts high on-treatment reactivity in clopidogrel low-responders. Multiple electrode aggregometry of whole blood from clopidogrel responders (A, mean AUC ±standard deviation after incubation with ADP: 280±86 AU/min) and low-responders (B, AUC with ADP 512±127 AU/min) normalized to the response induced by TRAP. The following agonist/antagonist concentrations were used: 32 µM TRAP, 6.5 µM ADP, 1, 50, or 100 µM serotonin, 10 µM ketanserin. ** p<0.01 and *** p<0.001 in ANOVA followed by Bonferroni's multiple comparison test.

We next asked whether clopidogrel responder status influenced these effects. Serotonin dose-dependently increased ADP-induced platelet aggregation in clopidogrel responders (Figure 3A): Mean ADP-induced aggregation was 33.7±1.3% and 60.0±3.1% in clopidogrel responders (n = 30) and clopidogrel low-responders (n = 12), respectively, and adding 50 µM serotonin increased ADP-induced aggregation to 40.9±2.0% (corresponding to an increase by 21%, p<0.01) and 62.6±5.2% (4% increase, not significant) in these groups. Higher serotonin concentrations (100 µM) further increased aggregation (to 48.2±2.0% in clopidogrel responders, 43% increase, p<0.001, and to 72.5±3.5% in clopidogrel low-responders, 21% increase, not significant). Therefore, serotonin enhanced platelet reactivity, especially in clopidogrel responders.

### Ketanserin improves platelet inhibition in clopidogrel low-responders

To examine whether anti-serotonergic treatment could reduce enhanced on-treatment platelet reactivity in clopidogrel low-responders, we blocked the main platelet serotonin receptor 5-HT2A with the standard antagonist ketanserin. Ketanserin reduced ADP-induced aggregation in clopidogrel low-responders only (from 60.0±3.1% to 37.4±3.5%, corresponding to a decrease by 37%, p<0.01, Figure 3B) and did not change ADP-induced aggregation in clopidogrel responders (Fig. 3A). In a subset of 13 CAD patients treated with ASA and clopidogrel (9 clopidogrel responders, 4 low-responders), sarpogrelate, a more selective 5-HT2A receptor antagonist, had a similar effect as ketanserin (not shown). These results indicate that residual platelet reactivity could be reduced in clopidogrel low-responders by inhibiting the 5-HT2A receptor.

### LTA reproduces the MEA results

**Figure 4:**
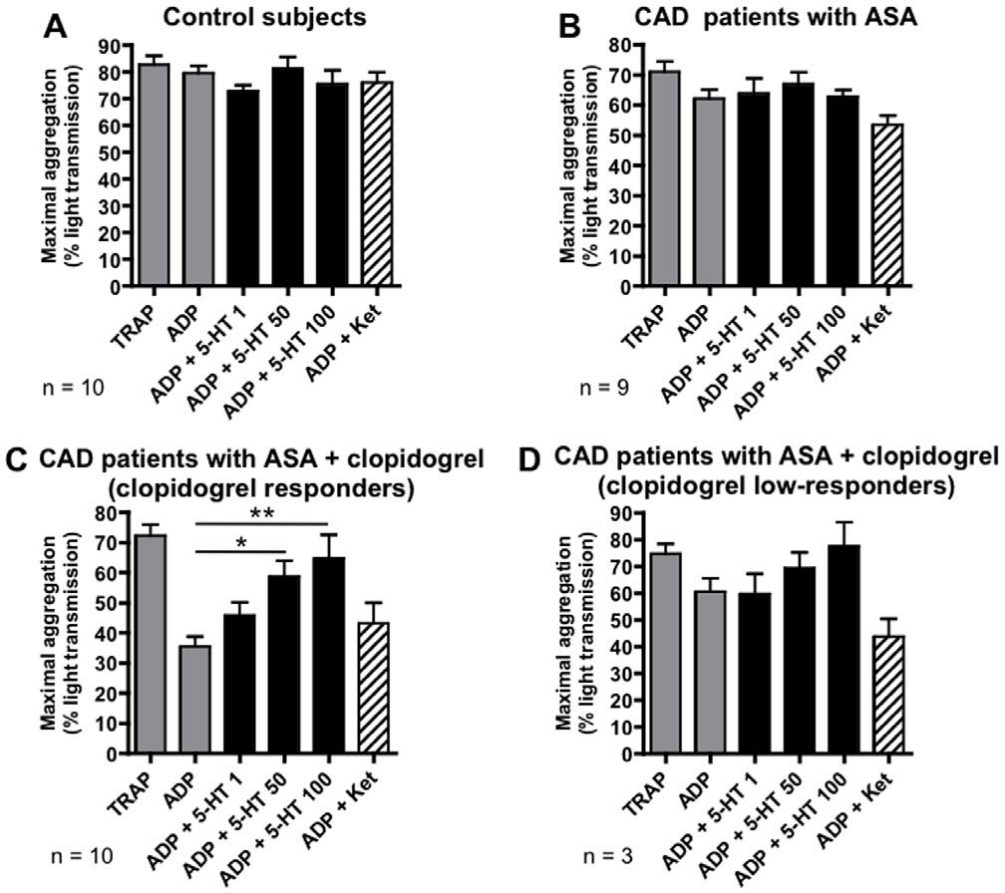
Confirmation with light transmission aggregometry. Maximal light transmission, i.e. platelet aggregation in platelet-rich plasma from healthy control subjects (A), CAD patients treated with ASA alone (B), and clopidogrel responders (C) and low-responders (D) among CAD patients treated with ASA and clopidogrel. The following agonist/antagonist concentrations were used: 32 µM TRAP, 6.5 µM ADP, 50 µM serotonin, 10 µM ketanserin. * p<0.05 and ** p<0.01 in ANOVA followed by Bonferroni's multiple comparison test.

LTA (with PRP) was performed as a gold standard to verify the MEA findings (whole blood) because to date, not all scientific groups have accepted whole blood impedance aggregometry as a standard platelet function test. In healthy control subjects TRAP treatment resulted in 81.9±2.5% light transmission, i.e. aggregation (Fig. 4A), compared to 5.8±0.8% with vehicle (not shown). In all CAD patients maximal aggregation was 72.1±2.1% after incubation with TRAP (Fig. 4B–D) and 9.6±1.9% with vehicle (not shown). Serotonin or ketanserin did not significantly change ADP-induced aggregation in healthy control subjects (Fig. 4A) or CAD patients treated with ASA alone (Fig. 4B). On the contrary, serotonin significantly increased ADP-induced platelet aggregation in PRP from clopidogrel responders from 35.5±3.2% to 58.8±5.1% with 50 µM serotonin (p<0.05) and to 64.8±7.7% with 100 µM serotonin (p<0.01, Fig. 4C). Clopidogrel low-responders showed a non-significant increase in ADP-induced aggregation with serotonin (Fig. 4D). Ketanserin did not significantly change ADP-induced aggregation in clopidogrel responders but induced a non-significant decrease by 46% in the 3 clopidogrel low-responders examined with LTA. In summary, LTA confirmed the MEA results (with a non-significant trend in the group with 3 clopidogrel low-responders).

### ADP-induced serotonin release is higher in clopidogrel low-responders

**Figure 5:**
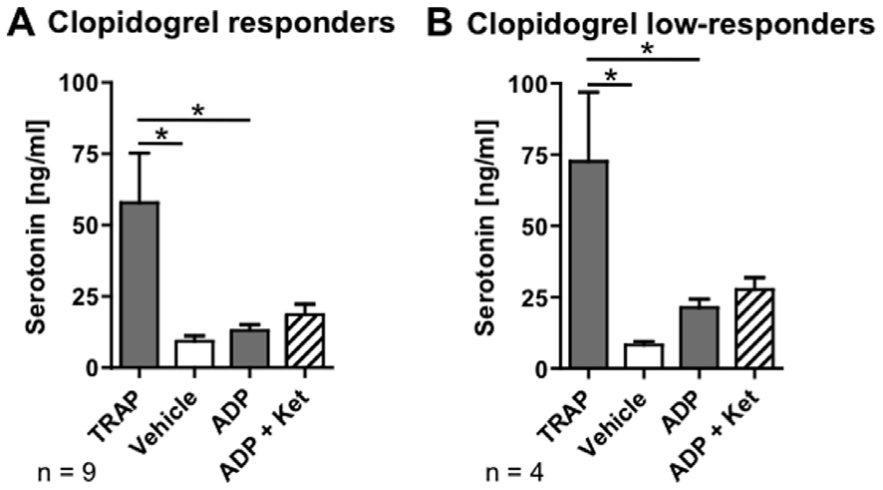
Serotonin release. Agonist-induced serotonin release was measured by high-sensitivity ELISA in a subset of 13 CAD patients treated with ASA and clopidogrel, among whom 9 were identified as clopidogrel responders (A) and 4 as low-responders (B). The following agonist/antagonist concentrations were used: 32 µM TRAP, 6.5 µM ADP, 10 µM ketanserin. * p<0.05 in ANOVA followed by Bonferroni's multiple comparison test.

In a subset of 13 CAD patients randomly chosen for measuring serotonin release, 4 were identified as clopidogrel low-responders. TRAP induced a significant serotonin release in both, responders and low-responders (Fig. 5). The TRAP-induced serotonin release was 12% higher in clopidogrel low-responders as compared to responders (not significant). ADP induced a 38% higher serotonin release in low-responders as compared to responders (not significant). A power calculation estimated a required sample size of 43 clopidogrel responders and 15 low-responders to reveal whether this difference is statistically significant.

## Discussion

This *in vitro* study with blood from 42 CAD patients treated with ASA and clopidogrel, 9 CAD patients treated with ASA only, and 10 healthy control subjects aimed at elucidating the effects of serotonergic and anti-serotonergic manipulation on platelet reactivity on top of standard treatment. While the standard of care treatment after coronary stent implantation includes dual antiplatelet therapy with ASA and a P2Y_12_ blocker, anti-serotonergic agents, namely 5-HT2A receptor blockers, were proposed as an adjunct in the newer concept of triple antiplatelet therapy.

Inhibition of the 5-HT2A receptor, which is the main serotonin receptor on platelets and arterial smooth muscle cells, with ketanserin or the newer, more selective agents sarpogrelate and APD791 prevented occlusive arterial thrombus formation in several studies [30], [31], [32], [33]. The group around Willerson established that ketanserin inhibits serotonin-induced cyclic flow variations caused by platelet aggregation and vasoconstriction in a canine model of coronary artery stenosis and injury [34], [35], [36], [37], [38], [39]. The same group also showed that high levels of serotonin are released locally during percutaneous coronary intervention and that serotonin antagonism can enhance coronary blood flow in patients with CAD [24], [40]. Sarpogrelate reduced restenosis after coronary stenting in a Japanese study of 79 patients with stable angina [41]. A phase III randomized trial with 1,510 patients showed non-inferiority of sarpogrelate to ASA in secondary prevention after stroke [42]. In Japan, sarpogrelate was approved for the treatment of peripheral arterial disease in 1993. We found recently that stimulation of 5-HT2A receptor induced shedding of the adhesion receptor GPIbα from the platelet surface [43]. In the light of clopidogrel hypo-responsiveness and the quest for optimal treatment of CAD patients, the effects of serotonergic intervention in CAD patients treated with ASA and clopidogrel are a topic of clinical relevance. Currently, no clinical trials investigating serotonin antagonism on top of dual antiplatelet therapy after percutaneous coronary intervention are registered at clinicaltrials.gov.

We examined whole blood from CAD patients taking ASA and clopidogrel after coronary stent placement with MEA and found that 28.5% did not respond adequately to clopidogrel. This is in accordance with previously published studies applying similar platelet function testing [12], [44]. We found that in clopidogrel responders, additional serotonin dose-dependently increased ADP-induced aggregation, i.e. on-treatment platelet reactivity to ADP. This suggests, that the platelet helper-agonist status of serotonin can also be extended to CAD patients with dual antiplatelet therapy [18], [22].

Most interestingly, antagonizing the platelet serotonin receptor with the standard 5-HT2A receptor blocker ketanserin highly significantly improved platelet inhibition in clopidogrel low-responders. Ketanserin reduced ADP-induced aggregation in these patients approximately reaching the levels of clopidogrel responders. These results allow the hypothesis that clopidogrel hypo-responsiveness could be overcome with a serotonin receptor blocker. A possible explanation for ketanserin being effective in clopidogrel low-responders but not responders, is that ADP may induce relevant serotonin release in clopidogrel low-responders only, because P2Y_12_ is not sufficiently inhibited in these patients. Indeed, we saw an increase in serotonin release after incubation with ADP in clopidogrel low-responders that appeared higher than in responders. Evaluating whether this increase is statistically significant will require a larger sample size as the one chosen for this pilot study.

The fact that ketanserin did not reduce platelet aggregation beyond clopidogrel responder levels is suggestive for a favorable risk profile of triple antiplatelet therapy with anti-serotonergic agents: the risk for bleeding may not increase. More studies, especially investigating *in vivo* administration of serotonin blockers in addition to standard treatment, are needed to confirm these results. Several clinicial trials have demonstrated that clopidogel low-responder status is associated with an unfavorable clinical outcome (reviewed in [11], [44]). Future trials evaluating triple antiplatelet therapy with anti-serotonergic agents, such as sarpogrelate or APD791, should particularly address the question whether morbidity and mortality can be reduced with serotonin blockers. In addition to suggested beneficial effects on vascular tone, ischemic tolerance, prevention of coronary restenosis, and insulin resistance, based on our findings, we hypothesize that this strategy may particularly improve platelet inhibition without further increasing bleeding complications [45], [46], [47].

### Limitations

As a single-center study with pilot character, this investigation included only 42 CAD patients, 12 of whom were clopidogrel low-responders. Such a small sample size limits the generalization of our conclusions. Larger studies are warranted to confirm the findings. We used ketanserin to antagonize the 5-HT2A receptor *in vitro*, because it is considered a long-established standard substance. Clinical use of ketanserin in CAD was promising at first, but has been discontinued because of an unfavorable risk profile, including a possible association with the development of diabetes [30], [48]. Future trials should therefore focus on the aforementioned newer, more selective agents, one of which (sarpogrelate) showed similar results in a subset of samples in our study. While the MEA findings were largely reproduced with LTA, the improvement of platelet inhibition in clopidogrel low-responders with ketanserin did not reach statistical significance with LTA. Only 3 out of the 13 CAD patients who donated additional blood for LTA measurements showed clopidogrel hypo-responsiveness and we believe that the non-significance of this result was mainly due to the particularly small sample size in this group.

### Conclusions

This *in vitro* study demonstrates that serotonin increases residual platelet reactivity in patients who respond to ASA and clopidogrel after coronary stent placement. In clopidogrel low-responders, serotonin receptor antagonism improves platelet inhibition, almost reaching responder levels. Further investigation of triple antiplatelet therapy with anti-serotonergic agents to decrease elevated on-treatment platelet reactivity therefore appears promising.
